# Modulated turbulent convection: a benchmark model for large scale natural flows driven by diurnal heating

**DOI:** 10.1038/s41598-024-66882-5

**Published:** 2024-07-10

**Authors:** Pavel Urban, Tomáš Králík, Věra Musilová, Ladislav Skrbek

**Affiliations:** 1 The Czech Academy of Sciences, Institute of Scientific Instruments, Královopolská 147, 612 00 Brno, Czech Republic; 2 Faculty of Mathematics and Physics, Charles University, Ke Karlovu 3, 121 16 Prague, Czech Republic

**Keywords:** Thermal convection, Temperature modulation, Heat transport, Natural flows, Climate sciences, Planetary science, Astronomy and planetary science, Physics

## Abstract

Our life is strongly affected by turbulent convective flows, driven by time-dependent thermal forcing, especially diurnal heating of the Earth’s surface by the Sun. In a laboratory experiment, we investigate their analogues: We study complex and extraordinary properties of turbulent buoyancy driven flows generated due to periodic modulation of the temperature of the plates of a Rayleigh–Bénard cell, with amplitudes both smaller and larger than either the positive or negative mean temperature difference between the top and bottom. We probe the turbulent flow of our working fluid – cryogenic helium gas – using temperature sensors placed in the cell interior and embedded in its plates. We discuss spatial and temporal structure of the heat flow, generalize validity of Nusselt versus Rayleigh number scaling Nu $\propto$ Ra$^\gamma$ with $\gamma \approx {1/3}$ at very high Ra for modulated convection and argue that this system represents a benchmark model which helps us understand the energy budget of ocean currents or weather formation on Earth subject to diurnal Sun heating as well as similar natural flows on Earth-like planets.

## Introduction

**Figure 1:**
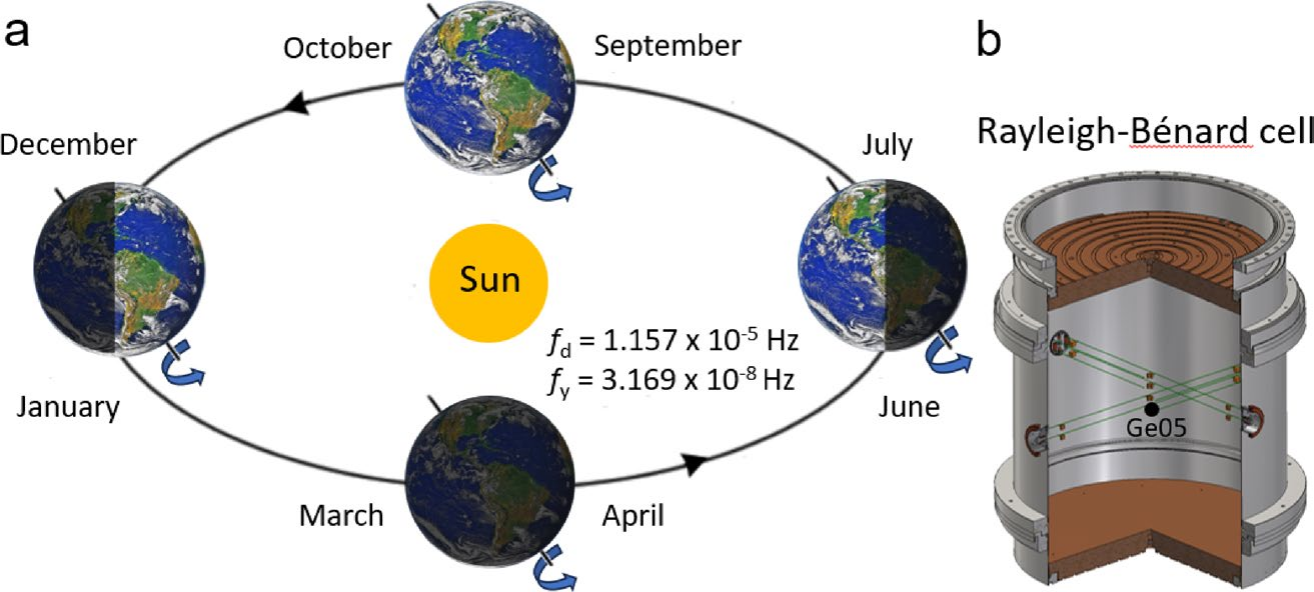
Diurnal heating by the Sun generates large scale convective flows adjacent to the Earth’s surface, modulated with daily, $f_{{\text{d}}}$, and yearly, $f_{{\text{y}}}$, frequencies (**a**). These flows can be modeled by periodically modulated Rayleigh–Bénard experiments performed in the cryogenic environment using the “thermally fast” experimental cell (**b**) equipped with various temperature sensors (the sensor Ge05 highlighted), as described in more detail in "Methods".

Thermal convection can be defined as the transfer of heat from one place to another due to the movement of fluid driven by buoyancy forces. A special case of thermal convection, Rayleigh–Bénard convection (RBC)^1,2^, occurs within a layer of Oberbeck-Boussinesq (OB) fluid (of constant physical properties except its density $\rho$ which depends linearly on temperature) between two horizontal, ideally conducting and laterally infinite top plate (temperature $T_{{\text{T}}}$) and bottom plate (temperature $T_{{\text{B}}}$; $T_{{\text{B}}}-T_{{\text{T}}}=\Delta T > 0$), separated by distance *L* in a gravitational field (acceleration due to gravity *g*). The steady-state RBC flow is fully characterized by the Rayleigh number ${\text{Ra}} = g \Delta T L^3\alpha /(\nu \kappa )$ and the Prandtl number, ${\text{Pr}} = \nu /\kappa$. Here $\alpha$, $\nu$ and $\kappa = \lambda /(\rho c)$ are, respectively, the thermal expansivity, kinematic viscosity, thermal diffusivity, thermal conductivity, density and specific heat at constant pressure of the working fluid. Laterally confined turbulent RBC, performed under stable laboratory conditions typically in cylindrical cells of diameter *D* and height *L* (aspect ratio $\Gamma =D/L$), belongs to the family of most frequently studied turbulent flows. Confined RBC serves as a tabletop model for steady-state large scale natural convective flows in the interior of stars such as the Sun^3^, as well as atmospheric^4,5^ or oceanic convective flows^6^ on Earth. These flows, such as complex atmospheric^7,8^ and oceanic flows^9,10^ are often driven by periodic thermal forcing, in particular, by diurnal heating of the Earth’s surface by the Sun (with daily, $f_{{\text{d}}}$, and yearly, $f_{{\text{y}}}$, frequencies), as schematically shown in Fig. 1a. Similar flows shape the weather of planets and their satellites^11–14^.

As for harmonically modulated RBC, several experimental and numerical studies of turbulent confined RBC flows driven by steady temperature difference $\Delta T$ with a weak superimposed sinusoidal temperature perturbation^15,16,19^ up to the case of fully periodic (i.e., of amplitude $\Delta T$) thermal drive^17,18,20^, have been performed. These studies have revealed interesting results, notably a significant enhancement of heat transfer efficiency of modulated turbulent RBC flow, numerically predicted, for moderate Ra $10^7 \le {\text{Ra}} \le 10^9$ by Yang et al.^20^ (see also earlier 2D numerical work of Raji^21^) and experimentally confirmed by Urban et al.^17^, for $10^8 \le {\text{Ra}} \le 3 \times 10^{12}$. Additionally, thermal waves have been detected by Urban et al.^18^, displaying constructive and destructive interference if generated coherently by periodically modulating the temperatures of the top and/or bottom plate of the RBC cell. Our aim is to extend these investigations with increased focus on results that have broad implications for the system being modeled, such as the solar diurnal cycle or other convection systems with fluctuating boundary temperatures.

**Figure 2:**
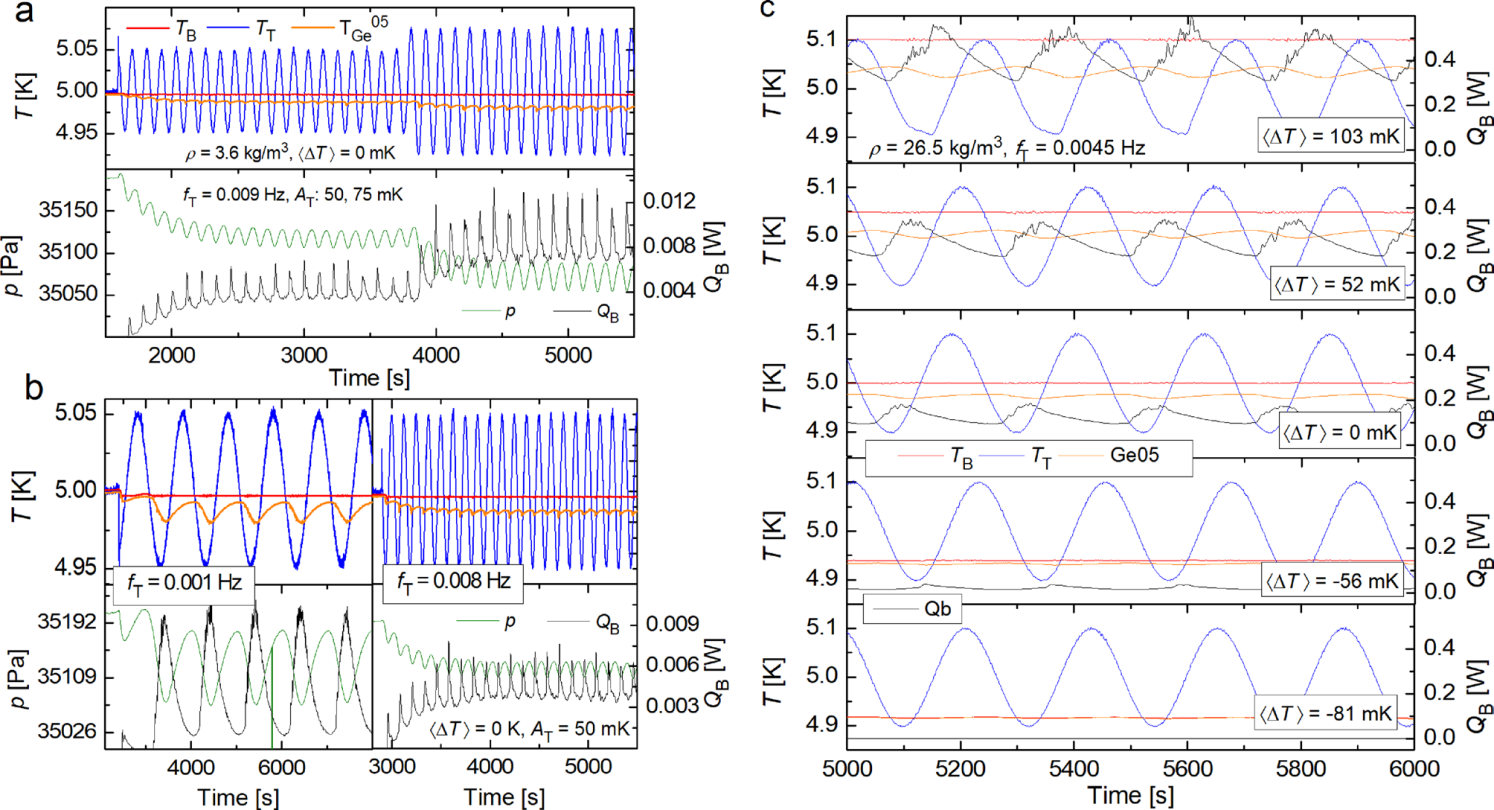
Panels (**a**, ** b**): Time records of temperatures $T_{{\text{B}}}(t)$ (red) and $T_{{\text{T}}}(t)$ (blue) measured at plates of the RBC cell, the temperature $T_\mathrm{{Ge}}^{05} (t)$ (orange) measured by the Ge-sensor No 5 in the bulk on the cell axis and of pressure in the RBC cell *p*(*t*) (green) together with heat input $Q_{{\text{B}}}(t)$ (black) delivered by the PID controller to the bottom plate in order to keep its temperature stable. The presence of temperature waves is most clearly indicated via time records of pressure *p*(*t*) in the RBC cell as described in the text. Panels (**c**): Time records of temperatures $T_{{\text{B}}}(t)$ (red), $T_{{\text{T}}}(t)$ (blue) and $T_\mathrm{{Ge}}^{05} (t)$ of the bulk (orange). Note the structure and phase shift of heat $Q_{{\text{B}}}(t)$ delivered to the bottom plate (black, right axis) in order to keep $T_{{\text{B}}}(t)$ stable while oscillating the top plate temperature as $T_{{\text{T}}}(t)=T_{{\text{T}}}+A_{{\text{T}}} \sin (2\pi f_{{\text{T}}})$, where $A_{{\text{T}}}$ and $f_{{\text{T}}}$ are the amplitude and the frequency of modulation. The individual panels correspond to various temperature gaps $\langle \Delta T(t) \rangle$ as indicated.

In order to understand the role of modulation for convective turbulent flows in its most naked form, we start with the basic case of the RBC flow with a zero mean temperature difference $\langle \Delta T(t) \rangle =0$ between its horizontal plates; the symbol $\langle ... \rangle$ means time averaging over several oscillation periods. For low Ra and Pr = 1 and 6, this case was previously studied in two dimensions (2D) numerically, by Wang et al.^22,23^. The temperature of either plate was sinusoidally oscillated. Despite no time-averaged temperature difference between the plates, the time-averaged Nusselt numbers, $\textrm{Nu}$ (specifying how many times more efficient is the buoyancy driven convective heat transfer in the working fluid than its molecular conduction), computed both at top and bottom plates, gave an upward time-averaged heat flux. This 2D numerical result calls for experimental 3D confirmation and extension to the more general case of finite $\langle \Delta T(t) \rangle$ of both signs while modulating the temperature of either plate with the amplitude ($A_{{\text{T}}}, A_{{\text{B}}}$) smaller or larger than $|\langle \Delta T(t) \rangle |$, especially at large, suitably defined Rayleigh numbers, relevant to highly turbulent periodically driven convective flows in Nature.

Here we present such a study, performed in a RBC cell shown in Fig. 1b, using a unique working fluid: cryogenic $^4$He gas with favorable, well-known^24^ and in situ tunable properties. Cryogenic conditions ($T \approx 5\,$K) are essential, as they provide highly conductive but low thermal capacity plates assuring uniform boundary conditions and little thermal load on the heat flow under study^25^; see "Methods" for details.

## Experimental results and their analysis

We start our investigation with the special case of a zero mean temperature difference $\langle \Delta T(t) \rangle =0$ between the plates. Specifically, we wait for thermal equilibrium between the top and bottom plates and force the top plate temperature to oscillate at fixed frequency $f_{{\text{T}}}$ as $T_{{\text{T}}}(t)= T_{{\text{T0}}} + A_\mathrm{{T}} \sin (2\pi f_{{\text{T}}}t)$ for various $A_\mathrm{{T}}$ while keeping the bottom plate temperature $T_{{\text{B}}}(t)=T_{{\text{B0}}}=T_{{\text{T0}}}$ stable. This is achieved by cooling the top plate using the gas heat exchange chamber above the RBC cell adjacent to the helium bath above it, of temperature 4.2 K, and using PID controllers delivering the appropriate, uniformly distributed heat fluxes to both plates. An example is shown in Fig. 2a, confirming that $\langle T_{{\text{T}}}(t) \rangle$ and $\langle T_{{\text{B}}}(t) \rangle$ remain constant and therefore also $\langle \Delta T(t) \rangle \approx 0$. Alternatively, as shown in Fig. 2b, we keep the modulation amplitude $A_\mathrm{{T}}$ fixed but increase in steps the modulation frequency, $f_{{\text{T}}}$.

The striking observation is the behavior of the mean temperature of the bulk, $\langle T_\mathrm{{Ge}}^n (t) \rangle$, directly measured by several ($n=1...12$) small Ge sensors placed in various positions inside the RBC cell (see^17,18^ and "Methods"). Within a wide range of $f_{{\text{T}}}$ the readings of all sensors in the bulk, $T_\mathrm{{Ge}}^n (t)$, are identical within the experimental accuracy of $\approx \pm 2\,$mK. We note that this feature occurred and was investigated in detail in our recent studies^17,18^ of harmonically modulated conventional RBC flows, with $\langle \Delta T(t) \rangle >0$ and $A_{{\text{T}}} \le \langle \Delta T(t) \rangle$ (or $A_{{\text{B}}} \le \langle \Delta T(t) \rangle$). Moreover, the temperature modulation of either plate resulted in generation of organized, inwards propagating imbalances^26^: thermal or heat waves ^27^, discernible here in the time records of $T_\mathrm{{Ge}}^n (t)$.

Why do all of the temperature readings $T_\mathrm{{Ge}}^n$ in the turbulent bulk of modulated RBC flow read as identical? The reason is that the vertical temperature gradient in the bulk of the RBC cell can be expressed via an effective heat conductivity $\lambda _\mathrm{{eff}}(z)=\kappa _\mathrm{{eff}}(z) c_{{\text{p}}} \rho$, where $\kappa _\mathrm{{eff}}(z)$ is the effective local thermal diffusivity of the working fluid. Experimental observations suggest that $\kappa _\mathrm{{eff}}(z)/\kappa$ (i.e., the local Nusselt number) in the turbulent bulk is very large and for $f_{{\text{T}}} \ll \tau _{ff}^{-1}$ the bulk temperature is almost uniform. Here $\tau _{ff}$ is the free fall time (about 1s in the here presented experiments), denoting how long it would take for a body of density equal to the density of the working fluid at the cold top plate temperature to fall from the top plate to the bottom plate, assuming no frictional forces, only the buoyancy force of the working fluid of lower density equal to that at the hot plate temperature. The situation is therefore, perhaps surprisingly, similar to conventional, steady-state high Ra RBC, where almost all of the temperature drop occurs over thin thermal boundary layers adjacent to plates. However, while in the steady RBC the boundary layers are symmetric under OB conditions, here, under periodic modulation, this symmetry is broken.

Upon a sudden change of $A_{{\text{T}}}$ or $f_{{\text{T}}}$, all $\langle T_\mathrm{{Ge}}^n(t) \rangle$ values relax together to a new stable mean value. This relaxation process of $\langle T_\mathrm{{Ge}}^n(t)\rangle$ towards its stable value occurs approximately exponentially, with a characteristic time of several oscillations. This is independently confirmed by the recorded pressure *p*(*t*) in the RBC cell, displaying oscillations which are in phase with all $T_\mathrm{{Ge}}^n (t)$ - fingerprints of heat waves propagating from the top plate. When *p*(*t*) is converted to temperature using the XHEPAK software^24^, under the assumption of constant density $\rho$ in the entire RBC cell, it agrees with the directly measured $T_\mathrm{{Ge}}^n(t)$ collapsing onto a single trace. We note that the data measured for different gas densities and modulation amplitudes look similar.

**Figure 3:**
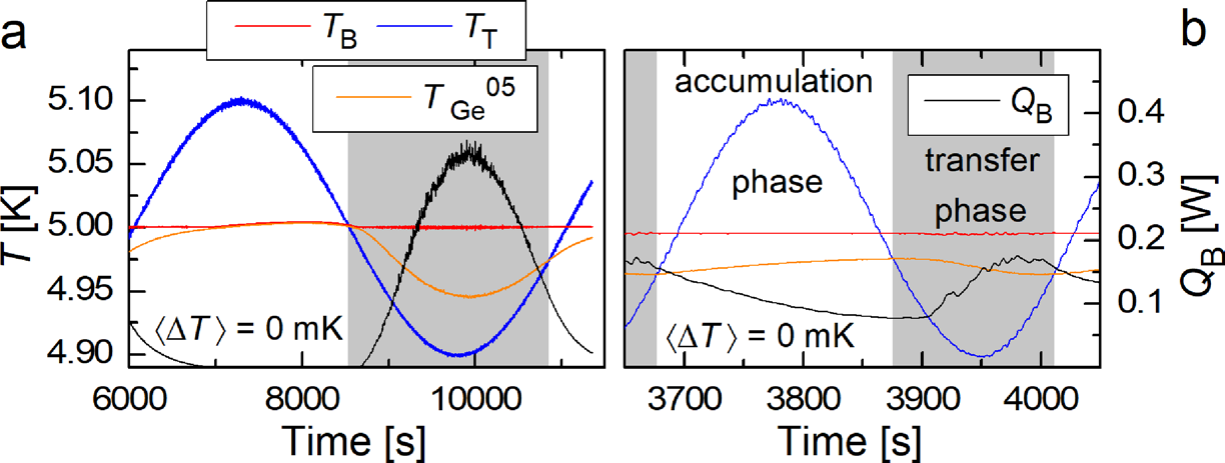
Recorded temperatures of the bottom (red) and top (blue) plates in comparison with the bulk temperature (orange – left axes and heat $Q_{{\text{B}}}(t)$ (black) delivered to the bottom plate--right axes. Intermittent (**a**) and continuous (**b**) heating cases are shown, with color-coded accumulation and transfer phases, as explained in the text.

**Figure 4:**
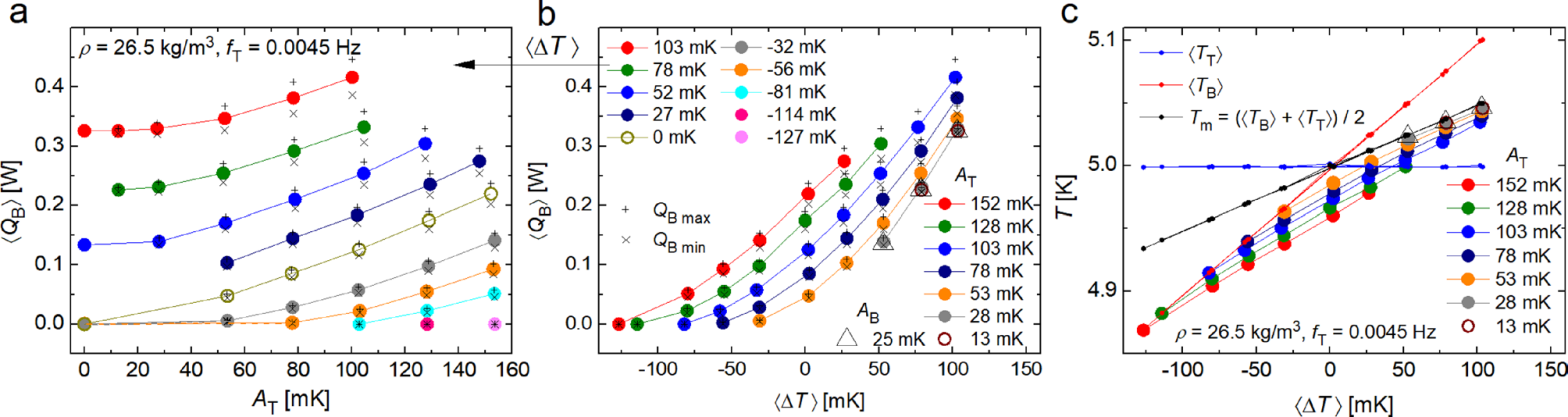
Time-averaged heating power $\langle Q_{{\text{B}}}(t) \rangle$ delivered to the bottom plate of the RBC cell in order to keep $T_{{\text{B}}}(t)$ stable, with $T_{{\text{T}}}$ modulated at frequency $f_{{\text{T}}}=0.0045\,$Hz, plotted (**a**) versus $A_\mathrm{{T}}$ for various $\langle \Delta T \rangle$ and (**b**) versus $\langle \Delta T \rangle$ for various $A_{{\text{T}}}$. The time averaging is done over entire oscillation periods (colored and empty circles), over transfer phases: $Q_{\mathrm{B~max}}$ ( + ) and over accumulation phases: $Q_{\mathrm{B~min}}$ ($\times$), defined in the text. Panel (**c**): The bulk temperature $\langle T_\mathrm{{Ge}}^{05} (t) \rangle$ (colored and empty circles) plotted versus the mean temperature difference between plates $\langle \Delta T \rangle = \langle T_{{\text{B}}} \rangle -\langle T_{{\text{T}}}(t) \rangle$ for various levels of temperature modulation $A_{{\text{T}}}$ as indicated, while keeping $\langle T_{{\text{T}}} \rangle =5\,$K stable. All solid lines serve as guides for the eye. The data series denoted in panels b and c by $\bigtriangleup$ are obtained by modulating the temperature of the bottom plate as $T_{{\text{B}}}(t)= T_{{\text{B0}}} + A_\mathrm{{B}} \sin (2\pi f_{{\text{B}}}t)$ for $f_{{\text{B}}}=0.0045\,$Hz; $A_\mathrm{{B}}=25\,$mK while keeping $T_{{\text{T}}}(t)$ stable.

In order to compare our laboratory experiment with natural large-scale modulated convective flows, we have performed more general experiments where, for constant $T_{{\text{B}}}(t)=T_\mathrm{{B0}}$ and oscillating $T_{{\text{T}}}(t)= T_{{\text{T0}}} + A_\mathrm{{T}} \sin (2\pi f_{{\text{T}}}t)$, the mean temperature difference $\langle \Delta T(t) \rangle$ between plates is changed in steps. The typical data are shown in Fig. 2c. They illustrate how $\langle \Delta T(t) \rangle$ and its time structure, $\Delta T(t) = T_{{\text{T0}}} +A_{{\text{T}}} \sin (2\pi f_{{\text{T}}}) -T_\mathrm{{B0}}$, controls the time-dependent heat $Q_{{\text{B}}}(t)$ that must be delivered by the PID controller to the bottom plate in order to keep its temperature stable.

Consider first the bottom panel of Fig. 2c, where balance among $\langle \Delta T(t) \rangle$, $A_{{\text{T}}}$ and $T_{{\text{B}}}$ has been found so that no heating, $\langle Q_{{\text{B}}}(t) \rangle$, is needed to maintain the statistically steady state. Here the effective convective heat transport takes place only over a small part of each thermal cycle. During the larger part of it, a much weaker molecular conduction acts in the downward direction. With no heat flow from the bottom plate to the bulk (i.e., for $Q_{{\text{B}}}(t) \approx 0$), there is no appreciable temperature drop between the plate and the bulk and thermal boundary layer does not develop. This thermal balance is, however, broken for all remaining panels of Fig. 2c.

Recorded $Q_{{\text{B}}}(t)$ values can be time-averaged in various ways: either over entire modulation periods, or over accumulation/transfer phases, defined as illustrated for $\langle \Delta T(t) \rangle =0$ in Fig. 3. While for sufficiently low $f_{{\text{T}}}$ the PID delivers heat to the bottom plate intermittently (a), for higher $f_{{\text{T}}}$ the heat delivery $Q_{{\text{B}}}(t)$ must be continuous (b). Each modulation period can be usefully divided in two phases. During the *accumulation phase*, the temperature of the bulk is lower than that of the top plate, so the heat transfer to the top plate cannot take place. At the same time, heat is continuously delivered to the cell via the bottom plate heater; during this stage heat is accumulating in the bulk and becomes transported to the top plate during the *transfer phase*. Non-zero $Q_{{\text{B}}}(t)$ must be delivered even at times when $T_{{\text{B}}}(t)<T_{{\text{T}}}(t)$ when heat flows from the colder bottom plate in the direction towards the hotter top plate and the system works as a heat pump. The described outcome does not qualitatively change when oscillating either $T_{{\text{B}}}(t)$ or $T_{{\text{T}}}(t)$. Application of this result to model diurnal heating of the Earth’s surface would mean an upward heat flow for the case of negative temperature gradient of the density of air or water (but downward heat flow for water between its freezing point and $\approx 4$ degrees Celsius).

Figure 4a,b display examples of time-averaged (over integer number of oscillating periods) heating powers $\langle Q_{{\text{B}}}(t) \rangle$ delivered to the bottom plate of the RBC cell while the top plate temperature is modulated as $A_{{\text{T}}} \sin (2 \pi f_{{\text{T}}})$, for various $\langle \Delta T(t) \rangle$. Also plotted are values of $\langle Q_{{\text{B}}}(t) \rangle$ time-averaged over transfer and accumulation phases. Without modulation, i.e., for $A_{{\text{T}}}(t)=0$ we arrive at the standard situation of conventional steady-state turbulent RBC. We emphasize that the topmost data series in Fig. 4a (satisfying the conditionn $A_{{\text{T}}} <\Delta T$) are in fair agreement with our recent study of modulated RBC^17^, confirming the significant enhancement of heat transfer efficiency in harmonically modulated RBC flow. The data series marked by open circles corresponds to the case $\langle \Delta T(t) \rangle =0$ between the plates. Rather surprisingly, a similar enhancement is also displayed in the case when $\langle T_{{\text{B}}}-T_{{\text{T}}}(t) \rangle < 0$, but it is shifted, roughly by $\langle \Delta T(t) \rangle$, to the right. Fig. 4b displays the time-averaged heating power $\langle Q_{{\text{B}}}(t) \rangle$ obtained for various modulation amplitudes $A_{{\text{T}}}$ versus time averaged temperature differences $\langle \Delta T(t) \rangle$. The data series denoted in panels b and c by $\bigtriangleup$ are obtained differently, namely by modulating the temperature of the bottom plate as $T_{{\text{B}}}(t)= T_{{\text{B0}}} + A_\mathrm{{B}} \sin (2\pi f_{{\text{B}}}t)$ for $f_{{\text{B}}}=0.0045\,$Hz; $A_\mathrm{{B}}=25\,$mK while keeping $T_{{\text{T}}}(t)$ stable. Still, there is an upward heat flow of amplitude that agrees with that observed when the upper plate is modulated, revealing the symmetry of the modulated RBC heat flow.

Figure  4c shows how the bulk temperature $\langle T_{{\text{Ge}}}^n(t) \rangle$, uniform over a wide range of modulation frequencies $f_{{\text{T}}}$, behaves in dependence of $\langle \Delta T(t) \rangle$ for various levels of temperature modulation $A_{{\text{T}}}$. As expected, for large negative $\langle \Delta T(t) \rangle$ approaching $-A_{{\text{T}}}$ the bulk temperature coincides with $T_{{\text{B}}}$, while in the limit of large positive $\langle \Delta T(t) \rangle$ it coincides with the mean temperature of the plates, $\langle T_{{\text{T}}}+T_{{\text{B}}}\rangle /2$, as in the conventional steady-state high Ra RBC.

## Theoretical model

Let us estimate the upward heat transfer of turbulent RBC flow under the following assumptions: the top plate temperature is modulated as $T_{{\text{T}}}(t) = T_\mathrm{{T0}} + A_{{\text{T}}} \sin (2 \pi f_{{\text{T}}}t)$, while $T_{{\text{B}}}(t)=T_{{\text{B0}}}$ is kept constant and that $f_{{\text{T}}}$ is so slow that the turbulent convective flow can be thought of as a sequence of consecutive steady states. The mean value $\langle \Delta T(t)\rangle = \langle \Delta T\rangle =T_\mathrm{{B0}}-\langle T_{{\text{T}}}(t) \rangle$ of the temperature difference between plates can be either positive or negative.

The convective heat flow will take place for the part of each modulation cycle when $T_{{\text{B}}} > T_{{\text{T}}}(t)$, (see Fig. 3a) with the instantaneous Rayleigh number defined in the usual way as ${\text{Ra}}(t) = g \frac{\alpha }{\nu \kappa }L^3 \Delta T_0^*(t)$. The time-dependent value $\Delta T_0^*(t)$ of the temperature difference between plates when turbulent convection takes place is either positive or set to zero where we suppose a weak heat transfer in the opposite direction by molecular diffusion, which we neglect. It therefore reads

1 $$\begin{aligned} \Delta T_0^*(t)=[\langle \Delta T\rangle -A_{{\text{T}}} \sin (2\pi f_{{\text{T}}}t)] \frac{{\text{sign}}[\langle \Delta T\rangle -A_{{\text{T}}} \sin (2 \pi f_{{\text{T}}}(t)]+1}{2}, \end{aligned}$$

where index 0 points to the low frequency model, ${\textrm{sign}}(x)_{x<0}=-1$; ${\textrm{sign}}(x)_{x=0}=0$, and ${\textrm{sign}}(x)_{x>0}=1$. Its mean value over entire period of modulation is

2 $$\begin{aligned} \langle \Delta T_0^*(t) \rangle =\frac{A_{{\text{T}}}}{4\pi } \int ^{2\pi }_0 \left[ \frac{\langle \Delta T \rangle }{A_{{\text{T}}}}-\sin \phi \right] \left[ {\text{sign}} \left( \frac{\langle \Delta T\rangle }{A_{{\text{T}}}}-\sin \phi \right) +1 \right] d\phi \,, \end{aligned}$$

where $$\langle \Delta T_0^*(t) \rangle \ge \langle \Delta T(t) \rangle$$; $$\langle \Delta T_0^*(t) \rangle = \langle \Delta T_0(t) \rangle$$ for $$A_{{\text{T}}} \le \langle \Delta T \rangle$$.

For conventional RBC flow with positive constant $\Delta T=T_{{\text{B}}}- T_{{\text{T}}}$ the conditions of this study would correspond to the range of high ${\text{Ra}}$ where scaling $\mathrm{{Nu(Ra)= \xi (Ra) Ra^{\gamma }}}$ with $\gamma \approx 1/3$, where $\xi ({\text{Ra}})$ is a numerical factor weakly decreasing with Ra, has been experimentally established in our earlier investigations^28–30^ in the same RBC cell^31^.

Heat to be delivered by the PID controller at any instant to the bottom plate of cross-section *S* and the height *L* is

3 $$\begin{aligned} Q_\mathrm{{B0}}(t)={\text{Nu}}(t)\frac{S \lambda \Delta T_0^*(t) }{L}= \frac{S \lambda }{L} \xi ({\text{Ra}}) \left( \frac{g \alpha }{\nu \kappa }L^3 \right) ^\gamma \Delta T_0^*(t)^{1+\gamma }\,, \end{aligned}$$

and the time-averaged heat delivered to the bottom becomes

4 $$\begin{aligned} \langle Q_\mathrm{{B0}}\rangle =\frac{S \lambda }{L} \left( \frac{g \alpha }{\nu \kappa } L^3 \right) ^{\gamma } \langle \xi ({\text{Ra}})\Delta T_0^*(t)^{1+\gamma } \rangle \,. \end{aligned}$$

We define ${\text{Ra}_0}=\frac{g \alpha }{\nu \kappa } L^3 \langle \Delta T_0^*(t) \rangle$ and ${\text{Nu}_0}= \frac{\langle Q_\mathrm{{B0}} \rangle }{\langle \Delta T_0^* \rangle } \frac{L}{S\lambda }$ based on the time-averaged heat flux $\langle Q_\mathrm{{B0}}\rangle$ and temperature drop $\langle \Delta T_0^*\rangle$; the zero index refers to $f_{{\text{T}}} \rightarrow 0$. Using equation (4) we get

5 $$\begin{aligned} {\text{Nu}_0}=\frac{\langle \xi ({\text{Ra}})\Delta T_0^*(t)^{1+\gamma } \rangle }{\langle \Delta T_0^*\rangle ^{1+\gamma } } {\text{Ra}_0}^\gamma = \xi _0 {\text{Ra}_0}^\gamma \,. \end{aligned}$$

**Figure 5:**
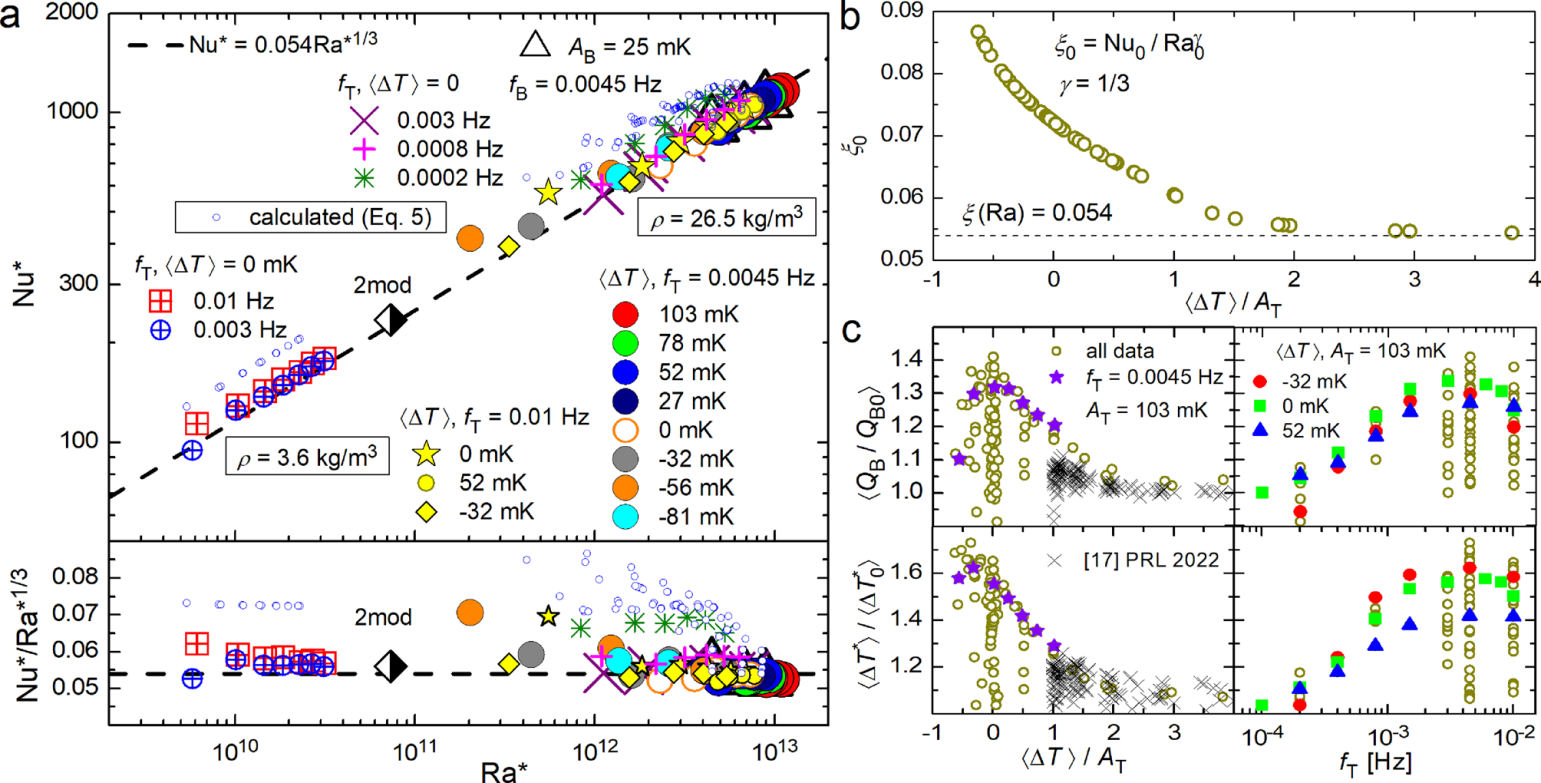
Panels (**a**): Effective $\mathrm{Nu^*=Nu^*(Ra^*)}$ scaling for highly turbulent strongly modulated convection (top) and the same data series (as indicated and described in more detail in the text) shown in the compensated form (bottom). The symbol denoted 2mod corresponds to the case of simultaneous modulation at two frequencies, as shown in Fig. 6. Panel (**b**): Prefactor $\xi _0({\text{Ra}})$ in equation (5) calculated for the particular case of power law scaling exponent $\gamma =1/3$, observed experimentally^28,29^ in the range of Ra of interest for unmodulated, steady-state RBC. Panels (**c**): Ratio of measured to calculated values of $\langle Q_{{\text{B}}}(t) \rangle /\langle Q_\mathrm{{B0}}\rangle$ and $\langle \Delta T^* \rangle /\langle \Delta T_0^*\rangle$ for various $A_{{\text{T}}}$ and $f_{{\text{T}}}$ plotted versus the controlling parameter $\langle \Delta T(t)\rangle /A_{{\text{T}}}$ (left) and versus $f_{{\text{T}}}$. The color-coded data series correspond to parameters as indicated; the crosses represent the data obtained in our recent study^17^, displaying the same decreasing tendency with increasing $\langle \Delta T(t)\rangle /A_{{\text{T}}}$.

The prefactor $\xi _0$ is plotted in Fig. 5b as a function of the ratio of the mean temperature drop between plates and the modulation amplitude: $\langle \Delta T\rangle /A_{{\text{T}}}$. This key parameter naturally controls the effect of modulation on heat transfer and fluid temperature in RBC. Above about two, $\xi _0$ approaches $\xi ({\text{Ra}})$ and the conventional unmodulated high ${\text{Ra}}$ scaling is recovered.

It is easy to modify the model for the case when the bottom plate temperature is modulated with the top plate temperature fixed and show that this leads to the same upward heat flow, as manifested in Fig. 4 by the data series denoted by $\bigtriangleup$.

## Generalization and impact of our experiments to natural large-scale, modulated convective flows

In order to generalize the description of the upward heat transfer efficiency through the RBC cell, we have to choose an appropriate key variable. Our choice (assuming temperature modulation of the top plate) is twice the temperature difference between the bottom plate and the uniform temperature of the turbulent bulk: $\Delta T^*= 2[T_{{\text{B}}} - T_\mathrm{{bulk}}(t)]$. It allows definition of effective $\mathrm{Nu^*}$ and $\mathrm{Ra^*}$, moreover, in the $A_{\text{T}} \rightarrow 0$ limit it coincides with the conventional definition of ${\text{Ra}}$, since under OB conditions $\Delta T^*=2[T_{{\text{B}}}(t) - T_\mathrm{{bulk}}(t)]_{A_{{\text{T}}} \rightarrow 0} = 2(T_{{\text{B}}}-T_{{\text{c}}})=T_{{\text{B}}}-T_{{\text{T}}}$. Importantly, introduction of $\Delta T^*$ (see equation (2) for the theoretical value at low frequencies) enables definition of Nusselt and Rayleigh numbers for modulated RBC even for negative values of $\langle T_{{\text{B}}}-T_{{\text{T}}} \rangle$. In view of the above discussion, any $T_{\text{Ge}}^n(t)$ can be used as $T_\mathrm{{bulk}}(t)$.

The ${\mathrm{Nu^*(Ra^*)}}$ scaling dependence for modulated RBC is plotted in Fig. 5a, containing both the experimental data as well as the data (small blue circles) calculated in the frame of the above simple model. Although it doesn’t take into account any possible frequency dependence, experimental data obtained for various $f_{{\text{T}}}$ can be used to explore it. The data obtained at low frequencies $f_{{\text{T}}}<0.0008$ Hz (green stars; $f_{{\text{T}}}=0.0002\,$Hz) approach points calculated for a sequence of RBC steady states in the limit $f_{{\text{T}}} \rightarrow 0$ (small blue circles). At higher modulating frequencies, all data (including those at negative values of $\Delta T/A_{{\text{T}}}$), nearly collapse on one line. It is remarkable that the ${\mathrm{Nu^*(Ra^*)}}$ scaling based on the temperature difference measured between the bottom plate and the bulk, $\langle \Delta T^*\rangle = 2\langle T_{{\text{B}}} - T_\mathrm{{bulk}}(t) \rangle$, follows the standard scaling of steady-state RBC.

We note that the threshold $0.0008\,$Hz between “low” and “high” frequencies corresponds approximately to the value $f_{{\text{S}}} =1$ of the non-dimensional frequency introduced in our earlier publication ^17^. Around this frequency, the phase shift between the sinusoidally modulated temperature of the plate and the response in the bulk of the fluid approaches the asymptotic value of $\pi /2$ (Fig. 3). The “${\mathrm{Nu^*(Ra^*)}}$ collapse” occurs together with enhancement of $\langle Q_{{\text{B}}} \rangle$ and $\langle \Delta T^*\rangle$ above the theoretical values of low frequency model $\langle Q_\mathrm{{B0}} \rangle$ and $\langle \Delta T_0^*\rangle$, see Fig. 5c. We see that experimentally observed frequency dependent enhancement reaches up to 40% in comparison with the low frequency model. Up to about 20% enhancement of heat transfer efficiency was indeed observed in our previous experiments limited with $\Delta T/ A_{{\text{T}}} \le 1$^17^. Additionally, we have also checked that this generalized scaling holds when the temperature of the bottom plate is modulated at frequency $f_{{\text{B}}}$ and amplitude $A_{\text{B}}$ while that of the top plate is kept constant; see the data series displayed as $\triangle$ in Fig. 5a.

**Figure 6:**
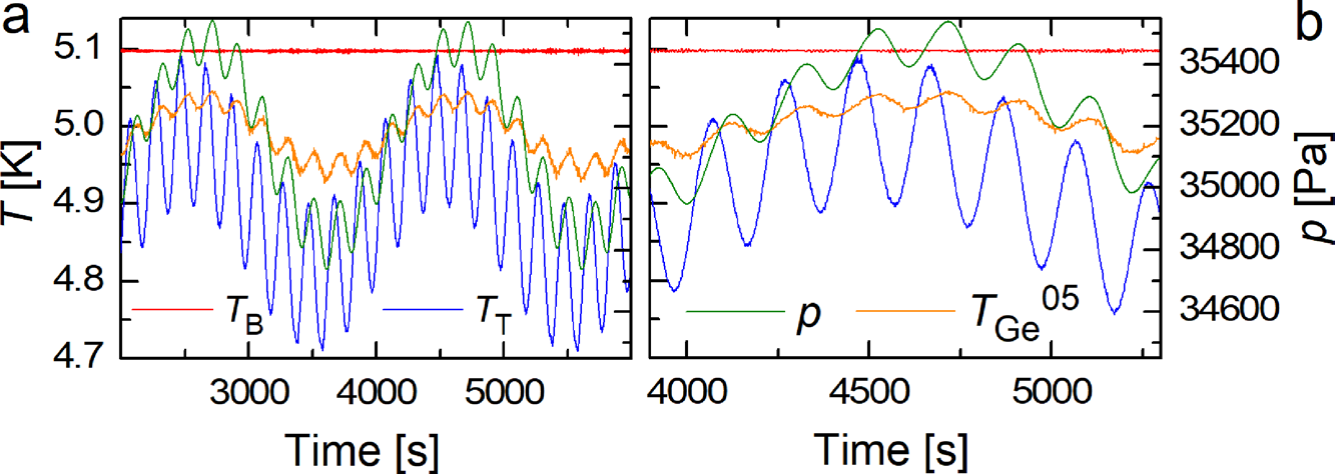
Time records of pressure, *p*, (right axes) and temperatures $T_{{\text{B}}}$, $T_{{\text{T}}}$ and $T_\mathrm{{Ge}}^{05} (t)$ (as indicated, left axes) for the case when the top plate temperature was modulated at two frequencies $f_{\text{T1}} \ll f_{\text{T2}}$ simultaneously. The phase shift between the averaged temperature of the plates $T_{{\text{m}}}(t)=(T_{{\text{T}}}(t)+T_{{\text{B}}})/2$ and the temperature of the bulk recorded by sensor $T_\mathrm{{Ge}}^{05} (t)$ (in phase with the pressure trace *p*(*t*)) is clearly visible at $f_{\text{T2}}$ (**a**) as well as at $f_{\text{T1}}$ (**b**).

In order to compare our laboratory experiment with the data taken directly from the natural large scale flows generated by diurnal heating of the Earth by Sun, we have performed a series of more directly related experiments. The diurnal heating illustrated in Fig. 1a is more closely modeled by modulation of the plate temperature at two frequencies $f_{\text{T1}} \ll f_{\text{T2}}$ simultaneously (corresponding to yearly and daily frequencies), as shown in Fig. 6a,b. Note the clearly visible phase shifts between the averaged temperature of the plates $T_{{\text{m}}}(t)=(T_{{\text{T}}}(t)+T_{{\text{B}}})/2$ and recorded $T_\mathrm{{Ge}}^5 (t)$ (in phase with the pressure *p*(*t*)). Figure  6a illustrates, for example, the analogy with the phase shift between the net surface heating power and subsurface temperature, as observed^10^ in hourly time series of properties of the diurnal shear layer, measured at the equatorial area of the Atlantic Ocean. Figure  6b demonstrates in detail the phase shift at lower $f_{\text{T1}}$ in analogy with the delay of about 1 month observed between the summer solstice and the maximum averaged day temperature in USA (depending on latitude), see Table 3.1^7^ or about 15 days, based on 39 years of records collected at ten meteorological stations in Hungary^8^. For these natural convective flows the estimated free fall time is at least an order of magnitude shorter in comparison with the temperature modulation period of one day for diurnal heating, therefore the suggested analogy is justified.

To conclude, using cryogenic helium gas as a working fluid, we have explored a class of buoyancy driven, convective turbulent flows generated in a thermally fast RBC cell by harmonic modulation of the temperature of one of its plates. We started with the case of the zero mean temperature difference between the plates and investigated the generated flow in dependence on the modulation amplitude $A_{{\text{T}}}$ ($A_{{\text{B}}}$) and frequency $f_{{\text{T}}}$ ($f_{{\text{B}}}$). Such a flow acts as a heat pump, independently on which of the plate’s temperature it is modulated.

Extended measurements for more general cases of both positive and negative non-zero mean temperature differences between the plates show that over wide ranges of $A_{{\text{T}}}$ and $f_{{\text{T}}}$ the turbulent bulk behaves almost uniformly, both spatially and temporally. For positive $\langle \Delta T \rangle = \langle T_{{\text{B}}}(t)-T_{{\text{T}}}(t) \rangle$, a previously claimed enhancement of heat transfer efficiency is confirmed and additionally found here for negative $\langle \Delta T \rangle$. These results have been obtained over a range of modulation frequencies $f_{{\text{T}}}$ spanning more than order of magnitude below the inverse free fall time of the corresponding steady-state RBC flow.

Our experiments allow the characterization of this class of turbulent, buoyancy driven, modulated convective flows via a generalized $\mathrm{Nu^*=Nu^*(Ra^*)}$ scaling, with Nusselt and Rayleigh numbers based on the time-averaged effective temperature difference $\Delta T^*= 2[T_{{\text{B}}} - T_\mathrm{{bulk}}(t)]$ (when $T_{{\text{T}}}$ is modulated). Our data confirm the robustness of the ${\text{Nu}}= \xi ({\text{Ra}}) {\text{Ra}}^\gamma$ scaling, with the exponent $\gamma$ approximately equal to 1/3 at high Ra. In the low-frequency limit, $\mathrm{Nu(Ra)}$ follows conventional scaling of steady-state RBC without modulation, when the key parameter $\langle \Delta T(t)\rangle /A_{{\text{T}}}$ controlling the heat transfer efficiency exceeds about two. Then the prefactor $\xi _0({\text{Ra}})$ approaches $\approx$0.054, recovering the scaling observed in unmodulated steady-state high Ra RBC^28,29^. At higher frequencies, the scaling ${\mathrm{Nu^*}}\approx 0.054{\mathrm{Ra^{*{1/3}}}}$ is approached for all values of $\langle \Delta T(t)\rangle /A_{{\text{T}}}$, including the negative ones.

The presented results ought to stimulate further investigation of modulated buoyancy driven turbulent flows. They have to be taken into account in consideration of various large scale natural buoyancy-driven flows due to diurnal heating by the Sun, i.e., for layers of fluids such as planetary atmospheres or layers of water on Earth, in which case the heat pumping effect can act thanks to the water anomaly around 4 degrees Celsius in both upwards and downwards directions.

## Methods

Our experiments are performed in the RBC cell $L=30$ cm tall and $D=30$ cm in inner diameter. Heat from its top plate is removed via the adjacent heat exchange chamber, seen in Fig. 1, to the liquid He vessel above. The top plate temperature is roughly set by pressure in the heat exchange chamber and more precisely tuned and modulated by the uniformly-distributed heater glued in the spiral groove on the upper side of the top plate. A similar heater delivers heat to the bottom plate. The cell in a deep cryogenic vacuum is surrounded by Al and Cu thermal shields attached to inner flange kept at the temperatures of liquid nitrogen and to the liquid helium vessel inside the cryostat. All twisted pairs of thin electric leads are thermally anchored at several temperature levels. This design allows to state that any parasitic heat leaks due to thermal radiation or thermal conduction are not an issue.

Under cryogenic conditions, the cell is “thermally fast” (see the detailed discussion in^25^): it faithfully and uniformly follows temperature modulations imposed to its plates. Contrary to the requirement of high heat capacity of the plates for studies of conventional steady-state RBC flow, for studies of harmonically modulated convective flows their heat capacity must be low, as it limits the modulation frequency due to the finite cooling rate on the negative slope of temperature modulation. The requirements for high thermal conductivity and low heat capacity are satisfied at cryogenic conditions by using 28 mm thick plates of thermally annealed copper, of thermal conductivity $\lambda _{{\text{p}}}=2210$ W m$^{-1}$K$^{-1}$ and thermal capacity $c_{{\text{p}}}=0.144$ J kg$^{-1}$K$^{-1}$ at cryogenic temperature $\approx 5$ K^31^. The required temperature modulation of either plate is achieved by applying suitable time-dependent heating delivered by home-made PID control schemes.

An accurate cryogenic thermometry is crucial. The cell is equipped with four calibrated Ge thermometers embedded in the middle and on the side of plates, used as primary thermometers. According to the Calibration Certificate (National Metrology Institute PTB Berlin) the uncertainty of the calibration of our GR-200A-1500 sensors is $\pm ~2$ mK in the range of 1.78 K to 10.45 K. Additional 12 small sensors^32,33^ inside the cell (see Ref.^17^ for their placement), calibrated by us against the primary four, directly measure the temperature in the turbulent core $T_{\text{Ge}}^n$ of the studied flow. Contemporary electronics allows us to detect temperature differences of order $10\,\mu$K, with a sensitivity of about $10^{-5}\, T_{\text{Ge}}^n$.

Experiments are performed around 5 K, using cryogenic helium gas with densities 26.5 kg/m$^{3}$ and 3.6 kg/m$^{3}$. For the former, in our RBC cell the onset of convection corresponds to undetectable value, less than 0.1 $\mu$K between plates; $A_\mathrm{{T}} \approx 100$ mK results in oscillations in Ra up to $10^{13}$.

### Supplementary Information

Supplementary Information.

## Data Availability

The key data (shown in Fig. 5) is provided in digital form within the supplementary information file. Additional data can be obtained upon reasonable request via e-mail urban@isibrno.cz.
