# Supplementary material for: Modulated turbulent convection: a benchmark model for large scale natural flows driven by diurnal heating
Source: Sci Rep. 2024 Jul 10;14:15987. doi: 10.1038/s41598-024-66882-5 (PMC11237114; doi:10.1038/s41598-024-66882-5)
Supplement: Supplementary file 1 — Supplementary Information. [file 41598_2024_66882_MOESM1_ESM.pdf]

Supplemental information for: Modulated turbulent convection: A model for large scale natural flows driven by diurnal heating

Pavel Urban[1], Tomáš Králík[1], Věra Musilová[1], and Ladislav Skrbek[2*]

[1]The Czech Academy of Sciences, Institute of Scientific Instruments, Královopolská 147, 612 00 Brno, Czech Republic
[2]Charles University, Faculty of Mathematics and Physics, Ke Karlovu 3, 121 16 Prague, Czech Republic
[*]Ladislav.Skrbek@mff.cuni.cz

ABSTRACT

Tables 1-5 below display the numerical values of the key experimental data shown as generalized Nu(Ra) scaling in Figure 5 of the main text. The values correspond to the symbols in Fig. 5 as indicated.

TABLE 1: Numerical values of experimental data shown in Fig. 5

quantity	ρ	f_T	$<\Delta T>$	Ra*	Nu*	symbol
unit	[kg/m^3]	[Hz]	[mK]	[-]	[-]	
	3.6	0.01	0	5.79E+09	94.6	blue circles with crosses
				1.01E+10	125	
				1.44E+10	137	
				1.85E+10	149	
				2.26E+10	160	
				2.66E+10	169	
				3.11E+10	176	
	3.6	0.003	0	6.14E+09	114	red squares with crosses
				1.04E+10	129	
				1.47E+10	143	
				1.85E+10	155	
				2.31E+10	163	
				2.72E+10	173	
				3.14E+10	179	

Figure 1

TABLE 2: Numerical values of experimental data shown in Fig. 5

quantity	ρ	f_{T1}	f_{T2}	$<\Delta T>$	Ra*	Nu*	symbol
unit	[kg/m^3]	[Hz]	[Hz]	[mK]	[-]	[-]	
	3.6	0.0005	0.005	0	7.35E+10	235	2-mod (diamonds)

Figure 2

TABLE 3: Numerical values of experimental data shown in Fig. 5

quantity	ρ	f_T	$<\Delta T>$	Ra*	Nu*	symbol
unit	[kg/m^3]	[Hz]	[mK]	[-]	[-]	
	26.5	0.003	0	4.35E+12	919	violet crosses
				3.16E+12	816	
				1.98E+12	702	
				1.12E+12	563	
				1.87E+12	663	
				6.04E+12	992	
				7.24E+12	1058	
	26.5	0.0008	0	4.17E+12	953	purple plus crosses
				3.16E+12	858	
				2.19E+12	736	
				1.09E+12	605	
				5.23E+12	1020	
				6.39E+12	1087	
	26.5	0.0002	0	3.26E+12	1029	green stars
				4.12E+12	1100	
				5.28E+12	1134	
				2.45E+12	914	
				1.67E+12	805	
				8.36E+11	626	

Figure 3

TABLE 4: Numerical values of experimental data shown in Fig. 5

quantity	ρ	f_T	$<\Delta T>$	Ra*	Nu*	symbol
unit	[kg/m^3]	[Hz]	[mK]	[-]	[-]	
	26.5	0.0045	103	1.10E+13	1166	red filled circles
				1.02E+13	1147	
				9.45E+12	1117	
				9.08E+12	1093	
				9.00E+12	1089	
	26.5	0.0045	78	9.36E+12	1122	green filled circles
				8.48E+12	1078	
				7.59E+12	1044	
				7.07E+12	1004	
				6.94E+12	1001	
	26.5	0.0045	52	7.71E+12	1065	blue filled circles
				8.93E+12	1117	
				6.62E+12	1019	
				5.66E+12	961	
				4.90E+12	891	
	26.5	0.0045	27	6.11E+12	999	navy filled circles
				8.55E+12	1093	
				7.59E+12	1049	
				5.17E+12	926	
				3.90E+12	868	
	26.5	0.0045	0	7.34E+12	1046	white filled dark yellow circles
				6.15E+12	986	
				4.67E+12	920	
				3.59E+12	806	
				2.31E+12	690	
	26.5	0.0045	-32	5.48E+12	954	gray filled cicrles
				4.09E+12	874	
				2.60E+12	794	
				1.61E+12	629	
				4.43E+11	452	
	26.5	0.0045	-56	4.05E+12	875	orange filled circles
				2.63E+12	781	
				1.23E+12	654	
				2.03E+11	415	
	26.5	0.0045	-81	2.58E+12	781	cyan filled circles
				1.36E+12	638	

Figure 4

TABLE 5: Numerical values of experimental data shown in Fig. 5

quantity	ρ	f_B	$<\Delta T>$	Ra*	Nu*	symbol
unit	[kg/m^3]	[Hz]	[mK]	[-]	[-]	
	26.5	0.0045	0	4.48E+12	914	white filled triangles
				6.78E+12	1000	
				8.88E+12	1086	

Figure 5
